# Efficacy of Shegan Mahuang Decoction for asthma: A systematic review and meta-analysis protocol

**DOI:** 10.1097/MD.0000000000017845

**Published:** 2019-11-01

**Authors:** Yuhua Zhao, Xiaoping Pang

**Affiliations:** a Zhejiang Xiaoshan Hospital, Hangzhou; b Taizhou Enze Medical Center (Group) Enze Hospital, Taizhou, Zhejiang, China.

**Keywords:** asthma, complementary medicine, evidence-based medicine, Shegan Mahuang Decoction, traditional Chinese medicine

## Abstract

**Background:**

Shegan Mahuang Decoction (SMD) was used widely for treatment of asthma in China; however, the clinical effect of SMD on asthma was not well concluded.

**Methods:**

Seven electronic databases (Medline, Cochrane library, EMBASE database, Chinese National Knowledge Infrastructure, Wanfang database, Chongqing VIP database, and Chinese Biomedicine database) will be searched for randomized controlled trials which meet the eligible criteria. Two reviewers will select studies and extract data independently. Risk of bias will be evaluated using modified Jadad score. Data synthesis will be carried out using RevMan 5.3. Sensitivity analysis and publication bias will also be investigated.

**Results:**

This systematic review and meta-analysis will review and synthesis current clinical evidence of SMD for the treatment of asthma.

**Conclusion:**

This systematic review and meta-analysis will provide high quality evidence of SMD for the treatment of asthma.

**Registration:**

PROSPERO CRD42019141810.

## 1 Introduction

Asthma is a common chronic inflammatory disease involving eosinophils, mast cells and T lymphocytes which affects up to 300 million people worldwide.^[1,2]^ It is mostly caused by genetic and environmental factors.^[3,4]^ The symptoms of asthma are always respiratory symptoms, included wheezing, shortness of breath, chest tightness, and cough.^[5,6]^ The objectives for asthma treatment are to minimize the symptom burden and the risk of adverse asthma outcomes. There are some pharmacological treatments such as β2 agonist, inhaled corticosteroids, leukotriene receptor antagonists, and theophylline.^[7,8]^ Although many symptoms can be controlled by steroids and β_2_ adrenergic agonist, there are a number of adverse effects which lead to limited adherence.^[9,10]^ New therapies with good efficiency and little adverse effect are needed.

Traditional Chinese medicine (TCM) nowadays offers an important alternative or complementary medication in many countries.^[11,12]^ Shegan Mahuang Decoction (SMD) is a classical prescription of TCM for asthma which mainly consisted of *Belamcanda chinensis* (Shegan) and *Ephedrae herba* (Mahuang).^[13]^*Belamcanda chinensis* and *E herba* is widely used in Korean, China, and Japan which have been used as traditional medicine for treatment of inflammation, asthma, sore throat, and tonsillitis.^[14]^ Modern studies have proved that chemical components of *B chinensis* and *E herba* can effectively ameliorate the progression of asthma.^[15–17]^ However, the clinical effect of SMD on asthma was not well concluded. We are writing this protocol to run a systematic review and meta-analysis which aims to synthesis the clinical evidence and evaluate the clinical efficiency of SMD for asthma.

## 2 Methods

This systemic review and meta-analysis protocol followed the Preferred Reporting Items for Systematic Review and Meta-Analysis Protocols (PRISMA-P) 2015 statement and was registered in PROSPERO (CRD42019141810).^[18]^ Ethic approval was not required because this review only involved published data.

### 2.1 Eligibility criteria

#### 2.1.1 Type of study

Only randomized controlled trials (RCTs) will be included for systematic review and meta-analysis.

#### 2.1.2 Participants

Patients with asthma diagnosed using any recognized diagnostic criteria will be included for study. Adults, adolescents, and elderly patients will all be included. However, asthma patients with other respiratory system disease will be excluded.

#### 2.1.3 Interventions

Patients treated with SMD alone or combined with other therapies will be included. The dose and administered frequency are not restricted.

#### 2.1.4 Comparison

Patients treated with placebo or any drugs other than SMD will be included.

#### 2.1.5 Publication data and language

Studies published before August 1, 2019 will be included. There is no restriction regarding the reporting language. The reviewers will seek professional interpreter help if the study is not published in Chinese or English.

### 2.2 Information source

Seven electronic databases will be searched for potential eligible studies. These databases are followed as: Medline, Cochrane library, EMBASE database, Chinese National Knowledge Infrastructure (CNKI), Wanfang database, Chongqing VIP database, and Chinese Biomedicine database.

### 2.3 Search strategy

A combination of following search terms will be used for electronic search: Shegan; *B chinensis*; *Belamcanda sinensis*; Mahuang; Ephedrae; Ephedra; Decoction; Tang; asthma. The combination will be modified to suit each database. Manual search will be carried out for any relevant studies, such as reference of yielded studies.

The literature search will be done by two reviewers (YHZ and XPP) independently, and any discrepancies will be solved by discussion. The literature search will be re-run before final data synthesis.

### 2.4 Study selection and data extraction

The search results of each database and manual search will be managed by reference managing software, NoteExpress. All duplicated records will be removed. Two reviewers (YHZ and XPP) will select the studies according to the eligible criteria. Reasons for any exclusion of studies will be recorded. The study selection process is independent for two reviewers (YHZ and XPP) and any discrepancies will be solved by discussion.

The following data items of eligible studies will be extracted: first author and published year, study duration and religion, randomization and blinding, number and age of participants, severity of illness, intervention of experimental groups and comparison group, primary and secondary outcomes. The extracted data will be managed using an Excel electronic table. The data extraction will be run by two reviewers (YHZ and XPP) independently and any discrepancies will be solved by discussion. If there is any missing data in the including study, a reviewer (YHZ) will contact the first author or corresponding author of the original study by E-mail.

### 2.5 Outcomes

#### 2.5.1 Primary outcome

The primary outcome of this meta-analysis is clinical effective rate. Clinical effect is defined as disappear or relief of asthma symptoms such as gasp, tachypnea, cough, and so on. The effect measure time is endpoint of original study.

#### 2.5.2 Secondary outcome

(1) Adverse event rate.
(2) Change of lung function.
(3) Quality of life.

### 2.6 Risk of bias assessment

The quality (risk of bias) of eligible studies will be assessed using modified Jadad score. The assessment will also be carried out by two reviewers (YHZ and XPP) independently and any discrepancies will be solved by discussion.

### 2.7 Data synthesis

The data will be reviewed before synthesis. Descriptive presentation of data will be carried out only if synthesis cannot be done. Data synthesis will be carried out using RevMan 5.3 software. Heterogeneity among studies will be assessed using *I*^2^ test. Mantel–Haenszel fixed effect model will be used if *I*^2^ is no more than 50%, or randomized effect model will be used. Odds ratio (OR) and 95% confidential interval (CI) will be calculated for primary outcome and other discontinuous results. Mean difference (MD) and 95% CI will be calculated for continuous outcomes.

If the included studies are sufficient, subgroup analysis will be done based on the following items:

(1) Combined therapies;
(2) Age of patients. Patients will be divided into adolescents (aged below 18), adult (aged between 18 and 65), elder patients (aged above 65);
(3) Severity of asthma.

### 2.8 Sensitivity analysis and publication bias

The sensitivity analysis will be carried out for the primary outcome. The data will be re-synthesized by excluding studies one by one, and the robustness of results will be evaluated.

Publication bias will be evaluated using funnel plot if included studies are more than 10.

### 2.9 Summary

The primary outcome will be summarized using the Grade of Recommendation Assessment, Development, and Evaluation approach.^[19]^ The finished systematic review and meta-analysis will be submitted to peer review journals.

## 3 Discussion

This systematic review and meta-analysis will be performed and written following the Preferred Reporting Items for Systematic Review and Meta-Analysis (PRISMA) 2009 statement.^[20]^ The evidence on effect of SMD for the treatment of asthma will be well concluded in this systematic review and meta-analysis for the first time. The results of this systematic review and meta-analysis would be helpful for physicians and asthma patients. However, the confidence of the results would be limited by the quality of include studies.

## Author contributions

**Funding acquisition:** Yuhua Zhao.

**Methodology:** Yuhua Zhao, Xiaoping Pang.

**Resources:** Yuhua Zhao.

**Software:** Xiaoping Pang.

**Supervision:** Yuhua Zhao.

**Writing – original draft:** Yuhua Zhao, Xiaoping Pang.

**Writing – review & editing:** Yuhua Zhao.
